# *Parvimonas micra* promotes colorectal tumorigenesis and is associated with prognosis of colorectal cancer patients

**DOI:** 10.1038/s41388-022-02395-7

**Published:** 2022-07-27

**Authors:** Liuyang Zhao, Xiang Zhang, Yunfei Zhou, Kaili Fu, Harry Cheuk-Hay Lau, Tommy Wai-Yiu Chun, Alvin Ho-Kwan Cheung, Olabisi Oluwabukola Coker, Hong Wei, William Ka-Kei Wu, Sunny Hei Wong, Joseph Jao-Yiu Sung, Ka Fai To, Jun Yu

**Affiliations:** 1 Department of Medicine and Therapeutics, Institute of Digestive Disease, State Key Laboratory of Digestive Disease, Li Ka Shing Institute of Health Sciences, CUHK-Shenzhen Research Institute, Shenzhen, The Chinese University of Hong Kong, Hong Kong SAR, China; 2 Department of Anatomical and Cellular Pathology, The Chinese University of Hong Kong, Hong Kong SAR, China; 3 Center of Precision Medicine, The First Affiliated Hospital, Sun Yat-sen University, Guangzhou, China; 4 Department of Anaesthesia and Intensive Care, The Chinese University of Hong Kong, Hong Kong SAR, China

**Keywords:** Prognostic markers, Colorectal cancer

## Abstract

Large-scale fecal shotgun metagenomic sequencing revealed the high abundance of *Parvimonas micra* in colorectal cancer (CRC) patients. We investigated the role and clinical significance of *P. micra* in colorectal tumorigenesis. The abundance of *P. micra* was examined in 309 fecal samples and 165 colon biopsy tissues of CRC patients and healthy subjects. *P. micra* was significantly enriched in fecal samples from 128 CRC patients compared to 181 healthy subjects (*P* < 0.0001); and in colon tissue biopsies from 52 CRC patients compared to 61 healthy subjects (*P* < 0.0001). Multivariate analysis showed that *P. micra* is an independent risk factor of poor survival in CRC patients (Hazard Ratio: 1.93). *P. micra* strain was isolated from feces of a CRC patient. *Apc*^min/+^ mice gavaged with *P. micra* showed significantly higher tumor burden and tumor load (both *P* < 0.01). Consistently, gavage of *P. micra* significantly promoted colonocyte proliferation in conventional mice, which was further confirmed by germ-free mice. *P. micra* colonization up-regulated genes involved in cell proliferation, stemness, angiogenesis and invasiveness/metastasis; and enhanced Th17 cells infiltration and expression of Th17 cells-secreted cytokines (Il-17, Il-22, and Il-23) in the colon of *Apc*^min/+^, conventional and germ-free mice. *P. micra*-conditioned medium significantly promoted the differentiation of CD4^+^ T cells to Th17 cells (IL-17^+^CD4^+^ phenotype) and enhanced the oncogenic Wnt signaling pathway. In conclusion, *P. micra* promoted colorectal tumorigenesis in mice by inducing colonocyte proliferation and altering Th17 immune response. *P. micra* may act as a prognostic biomarker for poor survival of CRC patients.

## Introduction

Colorectal cancer (CRC) is one of the leading causes of cancer-related deaths worldwide [1]. The progression of CRC involves a variety of factors including genetic mutations, epigenetic changes, and environmental alteration [2]. Accumulated evidence from us and others has suggested that the gut microbiota contributes to CRC development [3–5]. Gut commensal microbiota plays multiple roles in maintaining host health and inducing diseases [6]. A balanced microbiota could produce essential nutrients, prompt efficient host nutrient absorption, aid development of a mature and competent host immune system, and prevent pathogen colonization [7]. An unbalanced gut microbiota, commonly termed as dysbiosis, could result in inflammation, intestinal barrier failure, mucosal tissue damage, oncogene upregulation, and altered microenvironment, which all favor the development of CRC [8, 9]. Of note, the enrichment of opportunistic pathogenic bacteria in the gut microbiota of CRC patients has been constantly reported in previous studies which identified several individual species that contribute to CRC development. For instance, *Fusobacterium nucleatum* has been widely studied as a CRC-promoting microbe in both human and animal models by modulating tumor-immune microenvironment and autophagy [3, 10]. *Peptostreptococcus anaerobius* promotes colorectal carcinogenesis and modulates the tumor immune microenvironment by activating PI3K/Akt signaling via its PCWBR2 adhesin [5, 11]. Identifying microbial pathogens that are important contributors of colorectal tumorigenesis is imperative to the manipulation of gut microbiota for CRC prevention and treatment.

By conducting a large-scale meta-analysis of fecal shotgun metagenomic sequencing from four cohorts of different ethnicities (Chinese, French & German, Austrian and American), we identified the enrichment of *Parvimonas micra* in CRC patients compared to healthy controls [12, 13]. *P. micra*, formerly known as *Peptostreptococcus micros* or *Micromonas micros*, is a gram-positive, anaerobic and opportunistic pathogen commonly found in the human oral cavity [14]. *P. micra* is frequently isolated from a wide range of human infections, including orofacial odontogenic infections, periodontitis lesions, endodontic abscesses, and purulent pleurisy [15, 16]. Meanwhile, increasing evidence has reported the correlation between oral microbes and CRC [17], of which *P. micra* was found to be associated with the consensus molecular subtype 1 of CRC [18]. We therefore speculated that *P. micra* may be involved in the development of CRC. In this study, we investigated the functional importance of *P. micra* in multiple mouse models of CRC as well as its clinical implication. Our results altogether confirmed the CRC-promoting role of *P. micra* and its potential to be a prognostic marker for CRC patients.

## Results

### *P. micra* is significantly enriched in fecal samples and tissue biopsies of CRC patients

**Fig. 1:**
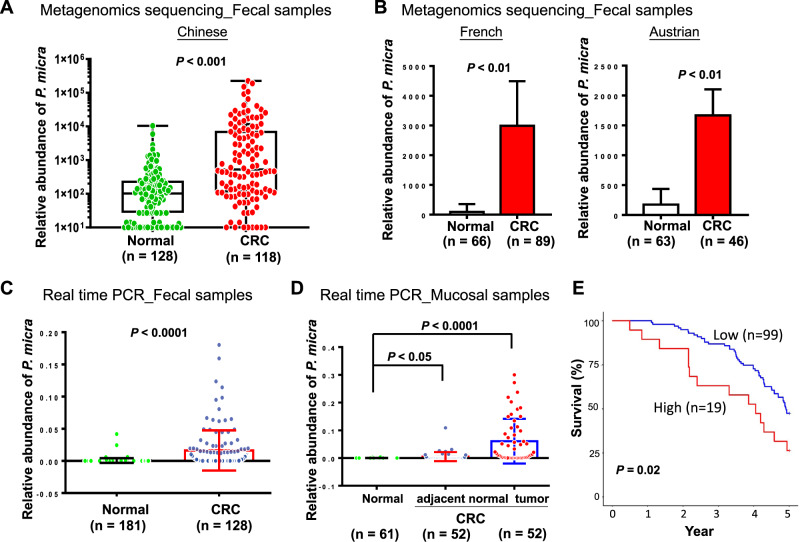
*P. micra* is enriched in stool and tissue samples of colorectal cancer (CRC) patients. **A** The level of *P. micra* in faecal samples of CRC patients (*n* = 118) and healthy subjects (*n* = 128) in Chinese by metagenomic sequencing; **B** *P. micra* level in stool samples from two public cohorts of French (CRC: *n* = 89 and healthy subjects: *n* = 66) and Austrian (CRC: *n* = 46; healthy subjects: *n* = 63) by metagenomic sequencing; **C** Real time qPCR analysis of faecal *P. micra* level in CRC patients (*n* = 128) and healthy subjects (*n* = 181) of Chinese cohort; **D** Real time qPCR analysis of paired CRC tumor and tumor-adjacent mucosal samples from CRC patients (*n* = 52) and mucosal samples from healthy subjects (*n* = 61); **E** Kaplan–Meier curves showed that high stool *P. micra* abundance was significantly associated with shortened survival in patients with CRC.

From our in-house fecal metagenomic cohort (118 CRC patients and 128 healthy subjects), we demonstrated that *P. micra* was significantly enriched in fecal samples of CRC patients compared to healthy subjects (*P* < 0.001) (Fig. 1A). The enrichment of *P. micra* in patients with CRC was validated in two independent metagenomic cohorts (French cohort: 89 CRC patients and 66 healthy subjects, *P* < 0.01; Austrian cohort: 46 CRC patients and 63 healthy subjects, *P* < 0.01) (Fig. 1B). To further verify the findings from metagenomic sequencing data, the abundance of *P. micra* was detected by real-time quantitative polymerase chain reaction (PCR) in additional fecal samples (CRC, *n* = 128; healthy subjects, *n* = 181). Our result revealed that *P. micra* was significantly enriched in CRC patients compared to healthy subjects (*P* < 0.0001) (Fig. 1C).

Gut mucosa microbiota is associated with colorectal carcinogenesis [19]. We therefore examined the abundance of *P. micra* in 61 mucosal samples of normal colon and 52 paired samples of colorectal tumor biopsies and adjacent normal mucosa. *P. micra* was significantly enriched in both tumor (*P* < 0.0001) and adjacent normal mucosa (*P* < 0.05) of CRC patients compared to healthy subjects, of which its abundance was the greatest in tumor tissues (Fig. 1D). Collectively, we confirmed the enrichment of *P. micra* in both fecal and mucosal samples of patients with CRC.

### *P. micra* is an independent predictor of poor outcome in CRC patients

We then analyzed the association of fecal *P. micra* abundance with clinicopathological features. There was no significant correlation between *P. micra* abundance and age, gender, or tumor-node-metastasis (TNM) stage (Table S1). Whereas *P. micra* was associated with increased risk of cancer-related death by univariate Cox regression analysis (Hazard Ratio (HR): 1.99, 95% confidence interval (CI): 1.10 to 3.60, *P* = 0.022; Table 1). After adjustment of confounding factors including age, gender and TNM stage, *P. micra* was identified as an independent risk factor of shortened 5-year survival in CRC by multivariate Cox regression analysis (HR: 1.93, 95% CI: 1.04 to 3.60, *P* = 0.037; Table 1). As shown in the Kaplan–Meier survival curves, patients with high abundance of *P. micra* had significantly shorter 5-year survival than those with low abundance of *P. micra (P* = 0.02, Fig. 1E). These results suggested that *P. micra* could be a predictor of poor prognosis in patients with CRC.

**Table 1:** Univariate and multivariate Cox regression analyses of potential poor prognostic factors for 118 in-house colorectal cancer patients with stool shot-gun metagenomic sequencing.

	Univariate Cox regression analysis		Multivariate Cox regression analysis	
Variables	HR (95% CI)	*p* value	HR (95% CI)	*p* value
Age				
<=65	1.32 (0.78 to 2.26)	0.30	1.33 (0.76 to 2.32)	0.33
>65	1.0		1.0	
Gender				
M	0.73 (0.45 to 1.19)	0.21		
F	1.0			
TNM clinical stage				
I & II	0.41 (0.25 to 0.67)	0.0004	0.44 (0.26 to 0.73)	0.0015
III & IV	1.0		1.0	
Obesity (Asian)				
No	1.0	0.10		
Yes	1.68 (0.91 to 3.09)			
Smoking status				
N0	1.0	0.22		
Yes	1.39 (0.82 to 2.35)			
Diabetes mellitus				
No	1.0	0.72		
Yes	1.10 (0.66 to 1.85)			
Oral prescription (4 weeks prior to sampling)				
No	1.0	0.79		
Yes	0.91 (0.47 to 1.79)			
*P. micra* abundance				
Low	1.0	0.022	1.0	0.037
High	1.99 (1.10 to 3.60)		1.93 (1.04 to 3.60)	

### *P. micra* promotes colorectal tumorigenesis in transgenic *Apc*^min/+^ mice

**Fig. 2:**
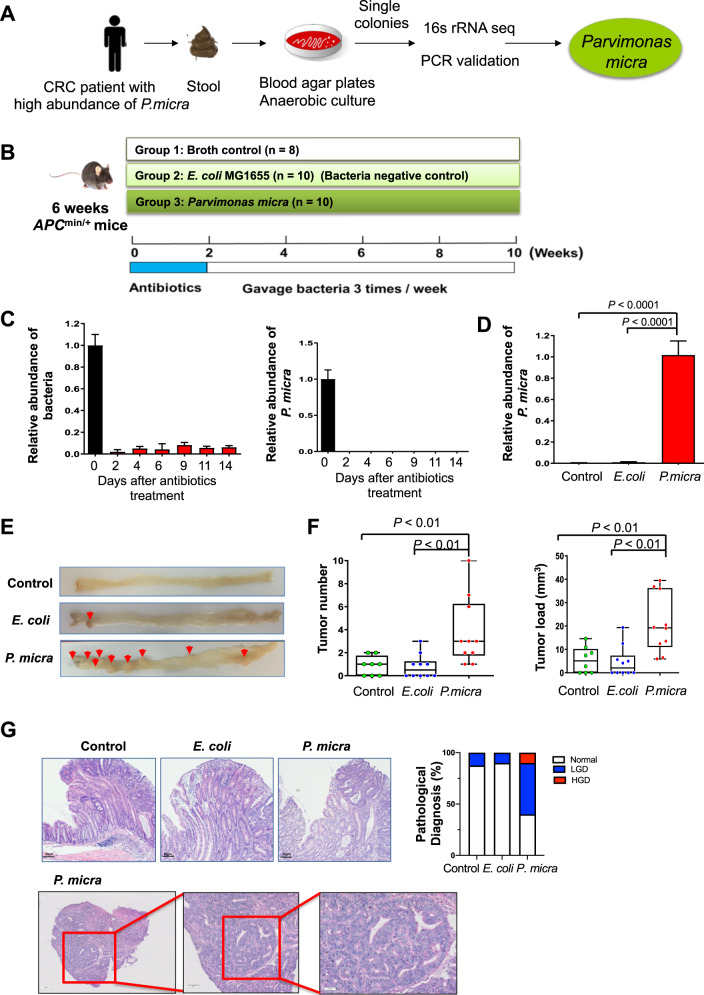
*P. micra* accelerates colonic tumorigenesis in *Apc*^min/+^ mice. **A** Isolation of *P. micra* in faecal sample of a CRC patient; **B** Schematic diagram showing the experimental design and timeline of *Apc*^min/+^ mice model (broth control group *n* = 8, *E. coli* MG1655 group *n* = 10 and *P. micra* group *n* = 10); **C** Faecal quantitation of total bacteria and *P. micra* in *Apc*^min/+^ mouse model after antibiotic treatment; **D** Relative abundance of *P. micra* in mice under different treatments; **E** Representative colonic morphologies in mice gavaged with *P. micra*, *E. coli* or broth control*;* **F** The colonic tumor numbers and loads of *Apc*^min/+^ mice under different treatments; **G** *P. micra* promoted the formation of colonic dysplasia. Representative histologic images of colon tissues of mice by H&E staining and statistical analysis of colon samples according to the histologic score. The low and high resolution histological images of the adenocarcinoma in *P. micra*-treated colon tissues were shown.

A strain of *P. micra* was successfully isolated from the fecal sample of a CRC patient (Fig. 2A and Fig. S1A). Chromatogram analysis showed that our isolated strain had nearly identical sequence to the 16 S ribosomal RNA (rRNA) gene sequence of *P. micra* in the NCBI RefSeq database (Fig. S1B). Growth dynamics our *P. micra* strain were shown in Fig. S2A. Given that *P. micra* was enriched in the colon mucosa of CRC patients (Fig. 1D), we co-cultured *P. micra* with HT-29, a human colon adenocarcinoma cell line with epithelial morphology. *P. micra* could adherent to HT-29 cells under anaerobic incubation compared to broth control (*P* < 0.0001), indicating the capability of *P. micra* to directly interacting with the gut epithelial cell (Fig. S2B).

To evaluate the role of *P. micra* in colorectal tumorigenesis, we used *Apc*^min/+^ mice which is a commonly used transgenic mouse model of spontaneous CRC (Fig. 2B). Before oral gavage of *P. micra*, the resident microbiota was depleted using a cocktail of broad-spectrum antibiotics (ampicillin 0.2 g/L, vancomycin 0.1 g/L, neomycin 0.2 g/L, and metronidazole 0.2 g/L) for 2 weeks (Fig. 2B). Microbiota depletion was confirmed by quantitative PCR which showed a 10-fold reduction of total bacterial DNA in mice feces (Fig. 2C). *Apc*^min/+^ mice were then orally gavaged with *P. micra*, a non-pathogenic *E. coli* strain MG1655, or broth control 3 times per week for 8 weeks (Fig. 2B). The fecal abundance of *P. micra* was increased after *P. micra* inoculation (Fig. 2D). *Apc*^min/+^ mice were euthanized after 10 weeks and the colon of each mouse was examined macroscopically and histologically. We observed significantly increased tumor number and tumor load in mice inoculated with *P. micra*, compared to mice with *E. coli* and broth control (both *P* < 0.01) (Fig. 2E, F). *P. micra-*gavaged *Apc*^min/+^ mice also showed significantly higher incidence of high-grade dysplasia (16.7%) and low-grade dysplasia (50.0%), compared to mice gavaged with *E. coli* or broth control (Fig. 2G). Collectively, these results indicated that *P. micra* plays a cancer-promoting role by accelerating colorectal tumorigenesis in *Apc*^min/+^ mice.

### *P. micra* promotes colonocyte proliferation in conventional C57BL/6 mice

**Fig. 3:**
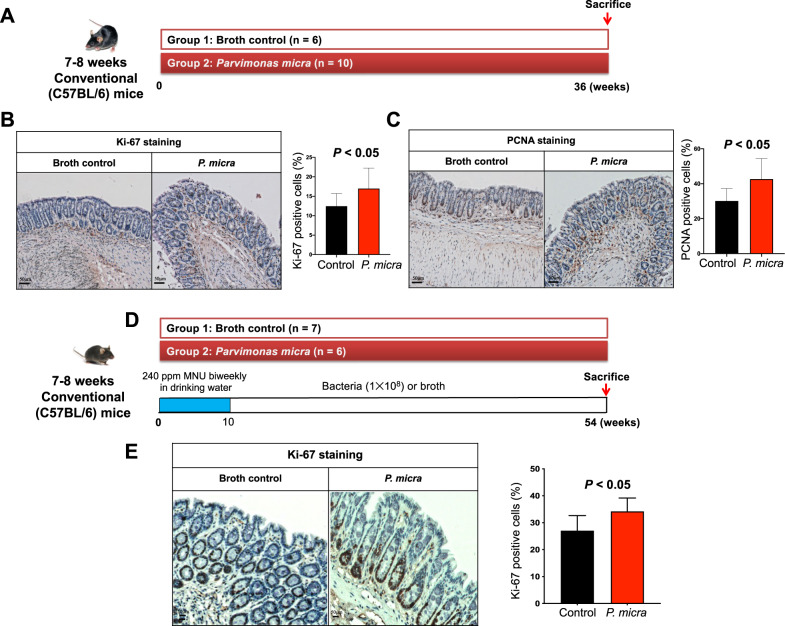
*P. micra* promotes colonocyte proliferation in conventional mice. **A** *P. micra* or broth control were gavaged to C57BL/6 conventional mice and harvested at 9 months; **B** Immunohistochemistry showing Ki-67-positive cells in the colon of conventional mice gavaged with *P. micra* or broth control*;* **C** Immunohistochemistry showing PCNA-positive cells in the colon of conventional mice gavaged with *P. micra* or broth control*;* **D** *P. micra* or broth control were gavaged to MNU-treated C57BL/6 conventional mice and harvested at 54 weeks; **E** Immunohistochemistry showing Ki-67-positive cells in the colon of MNU-treated conventional mice gavaged with *P. micra* or broth control.

To ascertain the role of *P. micra* in natural non-disease condition, we gavaged *P. micra* or broth control to conventional C57BL/6 mice without microbiota depletion, and mice were harvested after 36 weeks of gavage (Fig. 3A). Although no visible histological differences could be observed in mice colon (Fig. S3), increased proliferation of colon epithelial cells was identified in *P. micra*-gavaged mice compared to controls as indicated by a higher proportion of Ki-67 positive cells (*P*. micra *=* 17.0%, control = 12.5%; *P* < 0.05) (Fig. 3B) and proliferating cell nuclear antigen (PCNA) positive cells (*P. micra* = 42.7%, control = 30.1%; *P* < 0.05) (Fig. 3C).

We validated our in vivo results by establishing a carcinogen N-methyl-N-nitrosourea (MNU)-induced CRC mouse model (Fig. 3D). Consistently, the number of Ki-67 positive cells was significantly increased in *P. micra-*gavaged MNU mice (*P. micra* = 34.2%, control = 27.1%; *P* < 0.05) (Fig. 3E). Taken together, these results illustrated that *P. micra* promotes cell proliferation of colon epithelial cells.

### *P. micra* promotes colonocyte proliferation in germ-free mice

**Fig. 4:**
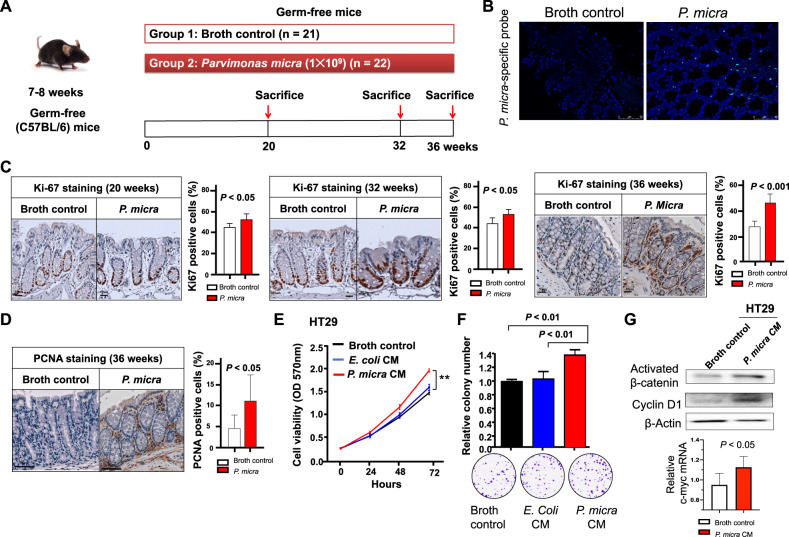
*P. micra* promotes colonocyte proliferation in germ-free mice. **A** Design of *P. micra* gavage experiment in germ-free mice; **B** Representative FISH images of colon tissue sections from BHI or *P. micra*-infected mice 36 weeks post-infection (Blue: nuclear; Green: *P. micra* probe*);* **C** Immunohistochemistry showing Ki-67-positive cells in the colon of germ-free mice at 20 weeks, 32 weeks, and 36 weeks after *P. micra* gavage. The proportion of Ki-67-positive cells was calculated in five fields of high-power microscopic field under microscopes. **D** Immunohistochemistry staining of PCNA protein in the colon of germ-free mice at 36 weeks after bacteria gavage; **E** MTT assay and **F** colony formation of HT29 cell line treated with broth control, *E. coli* conditioned medium (CM) and *P. micra* CM; **G** Western blot analysis of activated β-catenin and Cyclin D1 and real time PCR analysis of c-myc mRNA in HT29 cells treated with broth control or *P. micra* CM.

Germ-free mice were then used to examine the pure role of *P. micra* in the initiation and development of CRC. *P. micra* or broth control was given to germ-free mice, and mice were harvested at different time points of 20 weeks, 32 weeks, or 36 weeks (Fig. 4A). To examine the spatial distribution and intestinal localization of *P. micra*, we performed fluorescence in situ hybridization (FISH) using *P. micra* oligonucleotide probes on the colon tissues of germ-free mice. The germ-free status was confirmed by microbial FISH staining of the colon tissues using EUB338 universal bacterial probe (Fig. S4A), while the mono-colonization status of *P. micra* was accessed by co-staining of EUB338 universal bacterial probe and *P. micra*-specific probe. As shown in Fig. 4B, the intestinal colonization of *P. micra* was observed in germ-free mice gavaged with *P. micra* but not in the control mice. Whereas our results of FISH testified that germ-free mice were mono-colonized by a single bacterium (which is *P. micra*) without any other environmental microbes following *P. micra* gavage (Fig. S4B). Consistent to the findings from conventional mice, *P. micra* significantly induced colonocyte proliferation in the colon of *P. micra*-gavaged germ-free mice at 20 weeks (*P* < 0.05), 32 weeks (*P* < 0.05), and 36 weeks (*P* < 0.001), as compared to control mice (Fig. 4C). The ability of *P. micra* in promoting cell proliferation in the colon tissues of *P. micra*-gavaged germ-free mice was further confirmed by increased PCNA protein expression as compared with control mice (Fig. 4D). The findings from germ-free mice collectively suggested that *P. micra* could initiate CRC progression by inducing colonocyte proliferation.

### *P. micra* induces cell proliferation in vitro

To investigate its pro-tumorigenic functions, the conditioned medium of *P. micra* was treated with HT-29 CRC cell line. *P. micra*-conditioned medium significantly promoted proliferation of HT-29 cells compared with *E. coli*-conditioned medium and broth control, as evidenced by 3-(4,5-Dimethylthiazol-2-yl)-2,5-diphenyltetrazolium bromide （MTT） viability assay (Fig. 4E) and colony formation assay (Fig. 4F).

The oncogenic Wnt signaling pathway plays an important role in CRC. We therefore evaluated whether Wnt signaling pathway was involved in the development of *P. micra*-induced CRC. The treatment of *P. micra-*conditioned medium could significantly induce the protein levels of activated β-catenin and Cyclin D1 as well as the mRNA level of c-myc in HT-29 cells (Fig. 4G). These results hence revealed that *P. micra* could activate the Wnt/β-catenin signaling pathway in epithelial cells to contribute CRC progression.

### Altered expression of cell proliferation-related genes in *P. micra*-induced tumorigenesis

**Fig. 5:**
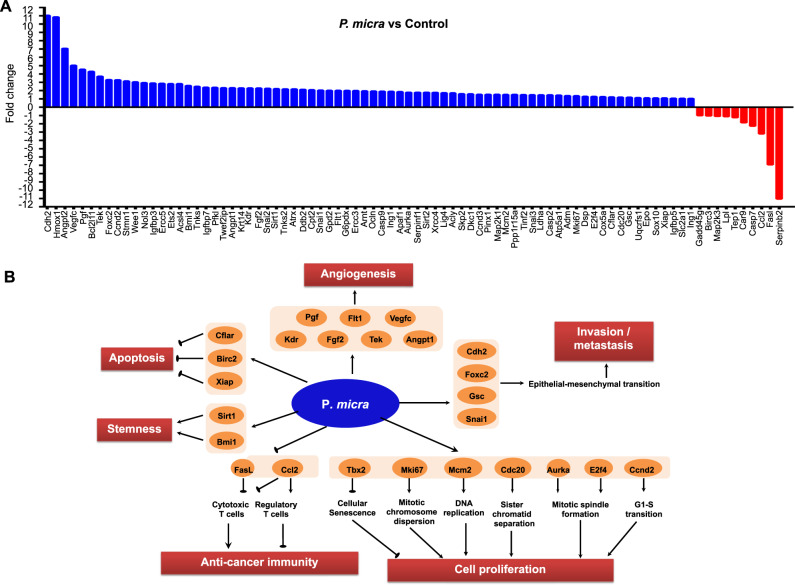
*P. micra* promotes oncogenic factors in germ-free mice. **A** Significant up-regulation of 74 transcripts and down-regulation of 10 transcripts by the Mouse Cancer Pathway Finder PCR Array in colon tissues of germ-free mice inoculated with *P. micra* for 32 weeks; **B** A systematic diagram showing major oncogenic pathways implicated by differentially expressed genes identified by the PCR array.

To uncover the mechanism underlying the pro-tumorigenic role of *P. micra*, we performed mouse Cancer Gene expression PCR Array on the colon tissues of germ-free mice (Fig. 5A). Genes involved in cell proliferation (*Tbx2, Mki67, Mcm2, Cdc20*), invasion and metastasis (*Cdh2, Foxc2, Snai1*), stemness (*Sirt1, Bmi1*), and angiogenesis (*Pgf, Tek, Angpt1, Fit1*) were observed to have more than 2-fold increase in expression in mice gavaged with *P. micra*, compared to the control mice (Fig. 5A, B). On the other hand, genes related to apoptotic pathways (*FasL, Casp7, Map2k3*) and DNA damage and repair (*Gadd45g*) were downregulated in *P. micra-*gavaged mice (Fig. 5A, B).

### *P. micra* induces Th17 immune cells infiltration

**Fig. 6:**
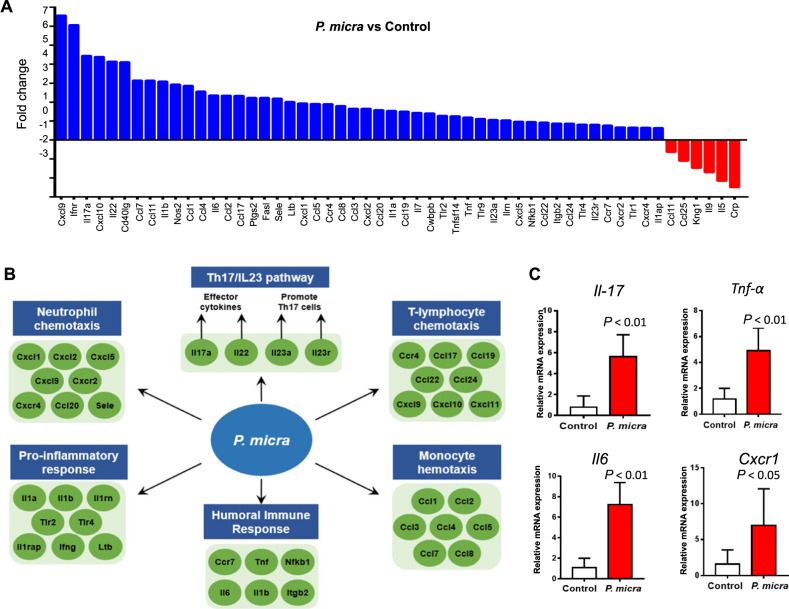
*P. micra* promotes inflammatory factors in germ-free mice. **A** Significant up-regulation of 48 transcripts and down-regulation of 10 transcripts by the Mouse Inflammatory Response and Autoimmunity PCR Array in the colon tissues of germ-free mice inoculated with *P. micra* for 32 weeks; **B** A systematic diagram showing major inflammatory pathways implicated by up-regulated genes identified by the PCR array; **C** qPCR validation was performed to confirm changes in expression of genes including Tnf, Il17a, Il16, and Cxcl1 in germ-free mice gavaged with *P. micra*.

The dysregulated immunity and chronic inflammation are known to be crucially involved in the development of gut microbiota-modulated CRC [6]. To explore immune response modulated by *P. micra*, we performed mouse Inflammatory Response and Autoimmunity PCR Array on colon tissues of *P. micra*-gavaged germ-free mice. Significant upregulation of 48 genes and downregulation of 6 genes were observed in germ-free mice after gavage with *P. micra* for 32 weeks, compared to the control mice (Fig. 6A). Differentially expressed genes included *interleukin* (*Il)-17a, Il-22*, and *Il-23a*, which encode 3 cytokines secreted by T helper (Th)-17 cells. The upregulated genes were involved in chemotaxis of various immune cells including neutrophil chemotaxis (*Cxcl1, Cxcl2, Cxcl5, Cxcl9, Cxcr2, Cxcr4, and Ccl20*), T-lymphocyte chemotaxis (*Ccr4, Ccl17, Ccl19, Ccl22, Ccl24, Cxcl9, Cxcl10, and Cxcl11*), and monocyte chemotaxis (*Ccl1, Ccl2, Ccl3, Ccl4, Ccl5, Ccl7, and Ccl8*) (Fig. 6B). Quantitative PCR confirmed the upregulated expressions of major pro-inflammatory cytokines including *Il-17a, Tnf-α, Il-6* and *Cxcr1* in the colon tissues of *P. micra-*gavaged mice (Fig. 6C), hence indicating that *P. micra* may contribute to dysregulated immunity by increasing secretion of inflammatory cytokines.

**Fig. 7:**
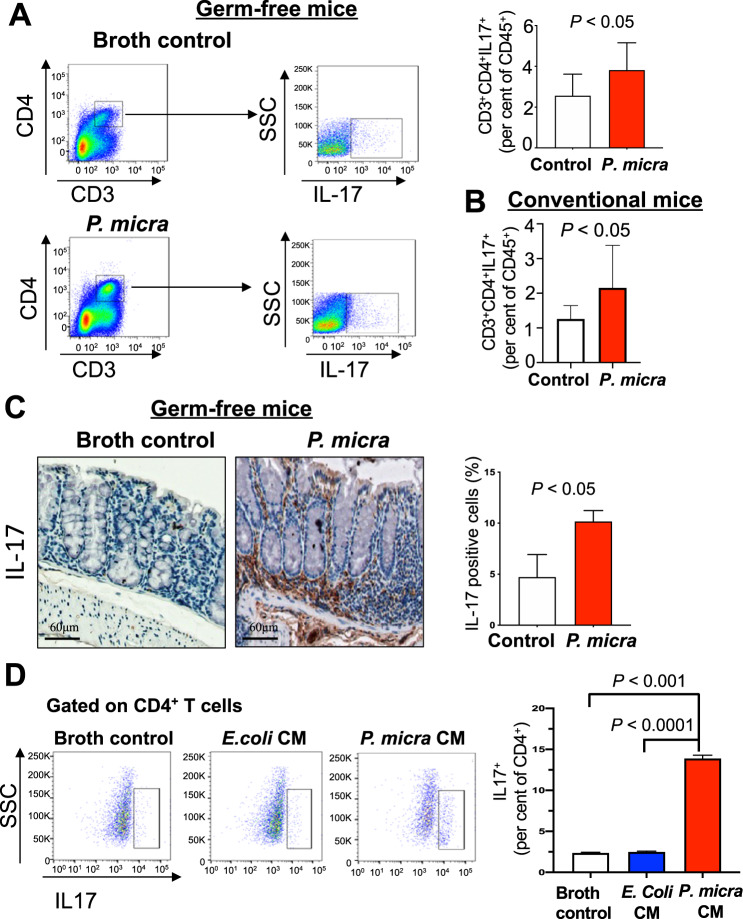
*P. micra* induced Th17 immune cells infiltration. **A** Flow cytometric analysis of Th17 cells in the colon of germ-free mice gavaged with *P. micra* or broth control*;* **B** Flow cytometric analysis of Th17 cells in the colon of C57BL/6 conventional mice gavaged with *P. micra* or broth control*;* **C** Immunohistochemistry staining of IL-17 in the colon of germ-free mice gavaged with *P. micra* or broth control; **D** The effect of *P. micra*-conditioned medium (CM) on Th17 cell differentiation compared to broth control or *E. coli* CM.

Intestinal Th17 cells are critical for maintaining tissue homeostasis [20]. As Th17-related cytokines including Il-17a, Il-22, and Il-23a were found to be upregulated (Fig. 6), we characterized Th17 immune cells in the colonic lamina of *P. micra*-gavaged germ-free mice. The number of colonic Th17 (CD4^+^Il-17^+^) immune cells was significantly higher in *P. micra-*gavaged germ-free mice (*P* < 0.05) compared to the corresponding control mice (Fig. 7A). The number of colonic Th17 cells was also significantly increased in *P. micra-*gavaged conventional mice (*P* < 0.05) (Fig. 7B and Fig. S5). Consistently, immunohistochemistry confirmed the enhanced colonic infiltration of Th17 cells in *P. micra*-gavaged germ-free mice (*P* < 0.05) (Fig. 7C).

To investigate if *P. micra* directly promotes the expansion of Th17 cells, we isolated CD4^+^ T cells from mouse spleen and treated them with *P. micra*-conditioned medium. Il-17 expression was evaluated by flow cytometry 5 days after the treatment. *P. micra*-conditioned medium significantly promoted cell differentiation to Il-17^+^CD4^+^ phenotype compared to broth control (*P* < 0.001) or *E. coli*-conditioned medium (*P* < 0.0001) (Fig. 7D). Taken together, these results suggested that the pro-tumorigenic effect of *P. micra* is associated with increased Th17 cell infiltration and differentiation in the colon, thereby promoting the establishment of a pro-inflammatory microenvironment which favors colorectal tumorigenesis.

## Discussion

We and others previously reported the enrichment of *P. micra* in fecal samples of CRC patients by shotgun metagenomic sequencing [13, 21]. In this study, increased abundance of *P. micra* in CRC patients were validated by multiple cohorts of fecal metagenomes and our in-house cohort of fecal samples. We confirmed that *P. micra* was significantly enriched in feces and tumor tissues of CRC patients compared with healthy subjects [22, 23]. Moreover, we identified that the fecal abundance of *P. micra* is an independent risk factor of poor survival in patients with CRC by multivariate Cox regression analysis (HR 1.93). In particular, the disease-free survival with high fecal abundance of *P. micra* was significantly shorter than those with low *P. micra* abundance. An accurate evaluation of prognosis is essential for clinicians to decide appropriate treatment for achieving maximized efficacy. Our results suggested that *P. micra* could serve as a new prognostic biomarker for CRC patients.

The role of *P. micra* in promoting colorectal tumorigenesis was elucidated in multiple mouse models including microbiota-depleted *Apc*^min/+^, conventional C57BL/6, and germ-free mice. We found that *P. micra* potentiates CRC progression as indicated by increased colon tumor number, tumor load, and incidence of histological dysplasia in *P. micra*-gavaged *Apc*^min/+^ mice. We further tested whether *P. micra* could contribute to CRC initiation by using conventional C57BL/6 mice without any carcinogen treatment. After gavage for 9 months, mice with *P. micra* inoculation had significant increased proliferation in colon epithelial cells which is an essential event for the development of precancerous colorectal lesion [24]. Such increase in epithelial cell proliferation was also observed in germ-free mice receiving a single gavage of *P. micra*, indicating that the pro-tumorigenic role of *P. micra* is independent from other gut microbes. In keeping with these findings, the expressions of genes related to cell proliferation, angiogenesis and invasion/metastasis were significantly upregulated in *P. micra-*gavaged mice compared to the control mice. These results collectively demonstrated that *P. micra* could accelerate colorectal tumorigenesis by promoting colonocyte proliferation.

In general, microbial communities in the oral cavity and gut are well-segregated due to the physical distance between these two regions, while the gastric acidity also limits the growth and further migration of translocated oral commensal microbes [25]. However, the oral microbiota could translocate to the intestinal mucosa when the oral-gut barrier is in dysfunction [26]. A previous study reported that the oral microbiota could overcome the physical barrier to invade and colonize in the gut, eventually reshaping the gut microbial community in germ-free mice [27]. In humans, many studies have revealed the presence and enrichment of oral commensals in patients with CRC, particularly *Fusobacterium nucleatum* which can promote colorectal tumorigenesis by generating a pro-inflammatory tumor immune microenvironment [10]. Given that physical barriers in the body are mostly likely weakened and impaired under pathological conditions, opportunistic pathogen including *P. micra* derived from oral cavity may move into the gut and potentially contribute to disease progression.

CRC-associated bacteria evoke colorectal tumorigenesis via various means. *F. nucleatum* modulates host immunity and tumor microenvironment by inducing mucin secretion and inflammatory cytokine TNF-α expression, while *P. anaerobius* promotes intracellular cholesterol biosynthesis to induce colon cell proliferation [5, 10]. Given the diverse mechanisms through which bacteria could promote tumorigenesis, we performed functional investigation to gain insights into the mechanisms employed by *P. micra* in CRC development. Our array analysis revealed that the pro-tumorigenic effect of *P. micra* is associated with altered immune responses and enhanced secretion of inflammatory cytokines in the gut. In particular, Th17-mediated cytokines, including Il17a, Il22, and Il23a, were significantly upregulated by *P. micra*. Il-23 and Il-17 signalling were found to be activated by microbial products and correlated with tumor growth [28]. The increased infiltration of Th17 cells in the colon tissues of *P. micra-*gavaged mice from multiple models was confirmed by flow cytometry and immunohistochemistry. In supporting our findings, previous studies have reported the roles of Th17-mediated inflammation in promoting tumor growth and progression in colorectal tumorigenesis [28, 29]. Taken together, our results suggested that *P. micra* promotes CRC development via enhancing Th17-mediated immune response.

In conclusion, we identified that *P. micra* is a novel pathogenic microbe to promote CRC initiation and development. *P. micra* drives colorectal tumorigenesis through enhancing Th17 immune responses and oncogenic factors in the colon. For clinical implication, the fecal abundance of *P. micra* may act as a prognostic marker of poor survival for CRC patients.

## Materials and methods

### Patient recruitment and samples collection

Fecal samples (128 CRC patients and 181 healthy subjects) were retrieved from the research stool bank, collected from individuals undergoing colonoscopy at Shaw Endoscopy Centre, Prince of Wales Hospital, The Chinese University of Hong Kong. The inclusion and exclusion criteria were described previously [30]. Mucosal biopsies (52 paired CRC tumor and tumor adjacent mucosal samples and 61 normal tissues) were obtained from individuals who had undergone standardized colonoscopy examinations previously [13, 19]. All samples were stored at −80 °C immediately after collection until further analysis. Metagenomic sequencing data of fecal samples from our in-house cohort (Chinese: 118 CRC patients and 128 healthy subjects) and two independent European cohorts (French: 89 CRC patients and 66 healthy subjects; Austrian: 46 CRC patients and 63 healthy subjects) were retrieved and analyzed. Written informed consent was obtained from all subjects and ethics was approved by The Joint Chinese University of Hong Kong - New Territories East Cluster Clinical Research Ethics Committee.

### Extraction of bacterial DNA from feces and tissues and *P. micra* quantification by quantitative polymerase chain reaction

200 mg of fecal samples were thawed on ice, and fecal DNA was extracted using ZR Faecal DNA MiniPrep Kit (Zymo Research, CA). The DNA quantity was determined using NanoDrop 2000c Spectrophotometer (Thermo Fisher Scientific, Waltham, MA). To extract bacterial DNA from human mucosal biopsies, glass beads (<100 µm, Sigma, St. Louis, MO) and QIAamp DNA Mini Kit (QIAGEN, Valencia, CAA) were used. 0.5 ng DNA was used for real time PCR analysis using Universal SYBR Greem Master (Roche, Risch-Rotkreuz, Switerland). The primers were listed in Table S2.

### Isolation of *P. micra* from faecal samples of CRC patients

Fecal samples from CRC patients with high relative abundance of *P. micra* were used for bacterial isolation. Samples were spread on horse blood agar plates and incubated in anaerobic chamber. The identity of the candidate colony (*P. micra* strain 512) was determined by gram staining and amplification of 16 S rRNA gene using universal primers targeting hypervariable regions V1-V4 and V6 of the 16 S rRNA gene, with further confirmation by *P. micra*-specific primers and Sanger sequencing. The bacteria were cultured in anaerobe basal broth (CM0957; Thermo Fisher Scientific) in anaerobic jar (Hardy Diagnostics, Santa Maria, CA). The anaerobic condition was created by the application of Anaerogen (AN0035; Thermo Fisher Scientific). Another strain (*P. micra* Smith) was bought from American Type Culture Collection (33270,ATCC, Manassas, VA).

### Animal experiments

Resident microbiota of male *Apc*^min/+^ mice (6 weeks old, *n* = 8–10/group) were depleted using a cocktail of broad-spectrum antibiotics (ampicillin 0.2 g/L, vancomycin 0.1 g/L, neomycin 0.2 g/L, and metronidazole 0.2 g/L) in drinking water for 2 weeks, before oral gavage with 1 × 10^8^ colony forming unit (CFU) of *P. micra, E. coli* MG1655 or broth control every 3 days for 8 weeks. Randomization was used to allocate the mice to different groups. *E. coli* MG1655 is a non-pathogenic human commensal intestinal bacterium which could not induce dysplasia [5, 31]. It was cultured in Brain Heart Infusion broth under aerobic condition. Mice were euthanized after 10 weeks, and their colon tissues were collected and examined.

In a separated model, conventional C57/BL6 male mice (7, 8 weeks old, *n* = 6–10/group) were gavaged with 1 × 10^8^ CFU of *P. micra* or broth control every 3 days for up to 9 months. Another group of mice (7, 8 weeks old) were administrated with 240 ppm MNU in the drinking water biweekly for 10 weeks to induce colon tumorigenesis. Mice were then randomly gavaged with 1 × 10^8^ CFU of *P. micra* or broth control and euthanized at week 54.

Germ-free male mice (7, 8 weeks old) were randomly gavaged with 1 × 10^8^ CFU of *P. micra* or broth control twice per week. Mice were euthanized at 20, 32, and 36 weeks respectively (*n* = 5–7/group). All animal experiments were performed in accordance with guidelines approved by the Animal Experimentation Ethics Committee of the Chinese University of Hong Kong.

### Histopathology

Mouse colon specimens were formalin-fixed and paraffin-embedded for histologic examination. Sections of 4 μm were stained with hematoxylin and eosin (H&E) for histologic diagnosis by an experienced pathologist (Dr. Alvin Ho-Kwan Cheung) who was blind to the group information. Dysplasia was defined according to the latest World Health Organization’s Classification of Tumors of the Digestive System.

### Immunohistochemistry staining

Paraffin-embedded tissues were used for analyzing the expressions of Ki-67 (#9449 S, Cell Signaling Technology, Danvers, MA), PCNA (#2586, Cell Signaling Technology) and IL-17 (ab79056, Cell Signaling Technology) by immunohistochemistry. Slides were incubated with primary antibody (1:100) at 4 °C overnight. Signals were developed with IHC HRP/DAB kit (Millipore, Burlington, MA). The proportion of Ki-67, PCNA, or IL-17-positive cells was determined by counting immunostaining-positive cells, as a percentage to the total number of nuclei in the field. At least 1000 cells were counted in 5 random microscopic fields.

### Fluorescence in situ hybridization

FISH-labeled *P. micra* Alexa Fluor 488-conjugated specific probe (GCCGCCGATCTAACCGCA) (Guangzhou exon biotechnology Co Ltd, Guangzhou, China) was used to detect *P. micra* colonization in paraffin-embedded sections. A Cy3-conjugated EUB338 universal bacterial probe (GCTGCCTCCCGTAGGAGT), as the positive control, was labelled with Spectrum-Red (Guangzhou exon biotechnology Co Ltd). 5 μm paraffin-embedded sections were hybridized in the hybridization buffer (1:50 in 25% hybridization buffer for *P. micra;* 1:50 in 45% hybridization buffer for EUB338 universal bacterial probe). After incubation overnight in a dark humid chamber at 40 °C, each slide was rinsed with sterile wash buffer and mounted with ProLong Gold Antifade Mountant with DAPI (Thermo Fisher Scientific).

### Cell culture

HT-29 colon cancer cell line was obtained from ATCC and grown in Dulbecco’s Modified Eagle’s Medium medium (Thermo Fisher Scientific) supplemented with 10% fetal bovine serum (Sigma-Aldrich). To obtain bacterial conditioned medium, *P. micra* CM or *E. coli* CM was centrifuged at 4500 g for 15 mins and filtered through 0.22 μm filter. The filtered medium was diluted to 12.5% CM with cell culture medium (DMEM + 10% FBS). For MTT assay, cells were seeded in 96-well plates (1000 cells/well). The cell culture medium was then replaced with 100 µL 12.5% *P. micra* CM or *E. coli* CM. Cell viability was examined by MTT assay. For colony formation assay, HT-29 cells were plated in 6-well plates and treated with *P. micra* CM or *E. coli* CM. After culturing for 10–14 days, cells were fixed with 70% ethanol and stained with 0.5% crystal violet solution. Colonies with more than 50 cells per colony were counted. All experiments were conducted in triplicates.

### Bacterial attachment assay

The bacterial attachment assay was performed as described previously described [11]; 1 × 10^6^ HT-29 colon cancer cells were grown in a 6-well plate and co-cultured with bacteria for 2 h (multiplicity of infection = 100) under anaerobic condition. After co-culture, cells were washed with phosphate-buffered saline (PBS) for three times and lysed with 1% Triton X-100 for 5 min. Cells with attached *P. micra* colonies were then recovered on horse blood agar plates under anaerobic condition, and the number of bacteria colonies that are adherent onto epithelial cells was counted after 4 days.

### Western blot

The total proteins isolated from cells or tissue samples and protein concentration were measured by DC Protein Assay (Bio-Rad Laboratories). 10 µg of proteins from each sample were separated on 10% SDS–PAGE and then transferred onto PVDF membranes. After blocking with 5% bovine serum albumin, blots were incubated with primary antibodies overnight at 4 °C. The primary antibodies used are as follows: activated β-catenin (05-665, Sigma), Cyclin D1 (#2922 S, Cell Signaling Technology), β-Actin (#4970, Cell Signaling Technology). Membranes were then incubated with secondary antibodies for 1 h at room temperature. Membranes were exposed to Pierce ECL Western Blotting Substrate (GE Healthcare). Band intensities were determined using ImageJ (National Institutes of Health).

### PCR array

Mouse Cancer Finder PCR array PM033ZC (Qiagen), including 84 genes representative of 9 different biological pathways involved in transformation and tumorigenesis, and Mouse Inflammatory Response and Autoimmunity PCR Array PAMM-077 (Qiagen), including 84 key genes of inflammatory cytokines and chemokines as well as their receptors, were performed according to the manufacturer’s protocol.

### Flow cytometry

Colon tissues were dissected and incubated in Hank’s balanced salt solution with 0.1 mg/mL collagenase D (Roche) and 50 U/mL DNase I (Roche) for 30 min at 37 °C. Lamina propria leukocytes was isolated as previously described [4]. Cells were then resuspended in staining solution (PBS with 2% fetal calf serum) for flow cytometry after staining with surface markers CD45 (103139, Biolegend, San Diego, CA), CD3 (100205, Biolegend), CD4 (100528, Biolegend), intracellular markers Il-17 (506903, Biolegend) and interferon (IFN)-γ (505806, Biolegend).

### CD4^+^ T cells isolation and treatment

CD4^+^ T cells were isolated from mouse spleen by EasySep™ Mouse CD4^+^ T Cell Isolation Kit (STEMCELL Technologies, Vancouver, Canada). CD4^+^ T cells were treated with *P. micra* CM or broth control, and flow cytometry analysis of Il-17 (506908, Biolegend) and CD4 (100528, Biolegend) was performed 5 days after treatment.

### Statistical analysis

All statistical tests were performed using GraphPad or R Software. Multiple group comparisons were analyzed by one-way analysis of variance (ANOVA) followed by post hoc tests (Bonferroni’s multiple comparisons test). The variables of the 2 sample groups were computed by Mann-Whitney *U* test or Student *t*-test. Two-way analysis of variance was performed to compare the difference in cell growth curves. The comparation of colonic dysplasia in different groups was analyzed by Chi-squared analysis. HR of survival associated with *P. micra* was estimated by using a univariate Cox regression model, and a multivariate Cox regression model was constructed to estimate the adjusted HR for high *P. micra* abundance. The overall survival in relation to *P. micra* abundance was evaluated by log-rank test. Data were presented as mean ± standard deviation. *P* < 0.05 was considered statistically significant.

## Supplementary information

Supplementary Methods

Figure S1

Figure S2

Figure S3

Figure S4

Figure S5

Table S1-S2
