# Supplementary material for: Parvimonas micra promotes colorectal tumorigenesis and is associated with prognosis of colorectal cancer patients
Source: Oncogene. 2022 Jul 27;41(36):4200–10. doi: 10.1038/s41388-022-02395-7 (PMC9439953; doi:10.1038/s41388-022-02395-7)
Supplement: Supplementary file 1 — Supplementary Methods [file 41388_2022_2395_MOESM1_ESM.docx]

## Supplementary Materials and Methods

**Cell viability and colony formation assay**

HT-29, a human colon adenocarcinoma cell line, was obtained from ATCC and grown in Dulbecco's Modified Eagle Medium (DMEM) supplemented with 10% fetal bovine serum (FBS). Cells were seeded in 96-well plate for 3-(4,5-Dimethylthiazol-2-yl)-2,5-Diphenyltetrazolium Bromide (MTT) assay and in 6-well plate for colony formation assay. Cultured *P. micra* and *E. coli* were centrifuged at 4,500 g for 15 min and filtered with a pore size of 0.22 µm to obtain the bacterial conditioned medium. The conditioned medium was diluted to 12.5% with cell culture medium (DMEM + 10% FBS), which was then used for cell culture. For MTT assay, 50 µl of MTT solution was added to each well and replaced with 100 µL of DMSO after 4 hours. The cell viability was determined by measuring absorbance at wavelengths of 570 nm. For colony formation assay, cells were fixed with methanol and stained with 0.5% crystal violet solution. Colonies with > 50 cells per colony were counted. All experiments were conducted three times in triplicates.

**RNA extraction and real-time PCR analysis**

Total RNA was extracted using TRIzol and transcribed into cDNA using a High Capacity cDNA Kit (Applied Biosystems, Foster City, CA). For quantitative PCR analysis, aliquots of cDNA were amplified using SYBR® Premix Ex Taq™ II (Takara Bio Inc, Japan) on LightCycler® 480 Instrument (Roche Diagnostics, Switzerland). Each sample was tested in triplicate. 2^-ΔΔCT^ method was employed to evaluate the fold change in gene expression level. 2^-ΔCT^ method was employed to determine the relative expression levels of corresponding genes. The sequences of mouse primers used were listed in **Table S2**.
