# Supplementary material for: Parvimonas micra promotes colorectal tumorigenesis and is associated with prognosis of colorectal cancer patients
Source: Oncogene. 2022 Jul 27;41(36):4200–10. doi: 10.1038/s41388-022-02395-7 (PMC9439953; doi:10.1038/s41388-022-02395-7)
Supplement: Supplementary file 2 — Figure S1 [file 41388_2022_2395_MOESM2_ESM.pdf]

Figure S1

A

No. of patients	Group	*P. Micra* Level in stool	No. of patients	Group	*P. Micra* Level in stool
M2-PK502A	Normal	0	M2-PKA10A	CRC	0.01928
M2-PK506A	Normal	0	M2-PKM113A	CRC	0
M2-PK-A60A	Normal	0	M2-PK008A	CRC	0.38442
M2-PK504A	Normal	0	M2-PK009A	CRC	0.00746
M2-PK514A	Normal	0	M2-PK010A	CRC	0.32358
M2-PK615A	Normal	0.31988	M2-PK011A	CRC	0
M2-PK532A	Normal	0	M2-PK015A	CRC	0.00158
M2-PK533A	Normal	0	M2-PK016A	CRC	1.03272
M2-PK543A	Normal	0	M2-PK017A	CRC	0
M2-PK548A	Normal	0	M2-PK018A	CRC	0

Stool samples

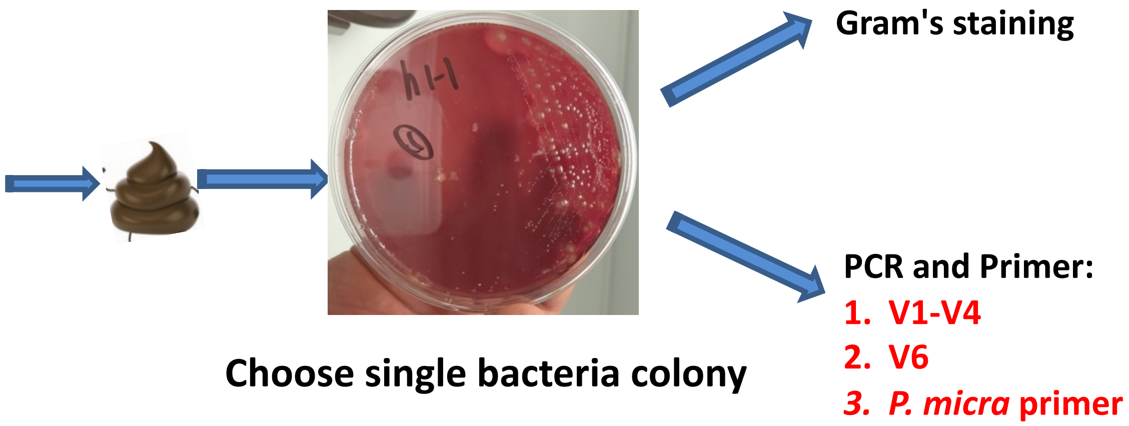

B

Sequences producing significant alignments:

Select: All None Selected:0

Alignments Download GenBank Graphics Distance tree of results

	Description	Max score	Total score	Query cover	E value	Ident	Accession
☐	Parvimonas micra strain KCOM 1037 chromosome, complete genome	713	2125	99%	0.0	100%	CP031971.1
☐	Uncultured Parvimonas sp. clone P26-34-T7 16S ribosomal RNA gene, partial sequence	713	713	99%	0.0	100%	KP294873.1
☐	Parvimonas micra strain NN01105 16S ribosomal RNA gene, partial sequence	713	713	99%	0.0	100%	KP944178.1
☐	Parvimonas micra strain 1002-10071 16S ribosomal RNA gene, partial sequence	713	713	99%	0.0	100%	KP944177.1
☐	Parvimonas micra strain KCOM 1535, complete genome	713	2141	99%	0.0	100%	CP009761.1

Alignments

Parvimonas micra strain KCOM 1037 chromosome, complete genome
Sequence ID: **CP031971.1** Length: 1661863 Number of Matches: 3
Range 1: 499277 to 499662

Score	Expect	Identities	Gaps	Strand	Frame
713 bits(386)	0.0()	386/386(100%)	0/386(0%)	Plus/Plus	

Features:

```
Query  1       AATTGATAAGTCCCTCGGGACAACTATACAGGTGGTGCATGGTTGTCGTCAGCTCGTGTC  60
Sbjct  499277  AATTGATAAGTCCCTCGGGACAACTATACAGGTGGTGCATGGTTGTCGTCAGCTCGTGTC  499336
Query  61      GTGAGATGTTGGGTTAAGTCCCGCAACGAGCGCAACCCTTATCTTCAGTTGCCAGCACGT  120
Sbjct  499337  GTGAGATGTTGGGTTAAGTCCCGCAACGAGCGCAACCCTTATCTTCAGTTGCCAGCACGT  499396
Query  121     AGAGGTGGGAACTCTGGAGAGACTGCCGATGACAAATCGGAGGAAGGTGGGGATGACGTC  180
Sbjct  499397  AGAGGTGGGAACTCTGGAGAGACTGCCGATGACAAATCGGAGGAAGGTGGGGATGACGTC  499456
Query  181     AAATCATCATGCCCTTTATGTCTTGGGCTACACACGTGCTACAATGGTTGGTACAACGAG  240
Sbjct  499457  AAATCATCATGCCCTTTATGTCTTGGGCTACACACGTGCTACAATGGTTGGTACAACGAG  499516
Query  241     AAGCGAGATAGAGATGTTAAGCGAAACTCTAAAAACCAATCTCAGTTCGGATTGTAGGCT  300
Sbjct  499517  AAGCGAGATAGAGATGTTAAGCGAAACTCTAAAAACCAATCTCAGTTCGGATTGTAGGCT  499576
Query  301     GCAACTCGCCTACATGAAGTCGGAGTTGCTAGTAATCGCGAATCAGAATGTCGCGGTGAA  360
Sbjct  499577  GCAACTCGCCTACATGAAGTCGGAGTTGCTAGTAATCGCGAATCAGAATGTCGCGGTGAA  499636
Query  361     TGCGTTCCCGGGTCTTGTACACACCG  386
Sbjct  499637  TGCGTTCCCGGGTCTTGTACACACCG  499662
```

Figure S1. Isolation and confirmation of *P. micra* colonies from CRC stools. (**A**) Selection of stool samples with comparatively high relative abundance of *P. micra* from CRC patients (M2-PK008A and M2-PK016A) and a control individual (M2-PK615A) for isolation of bacterial colonies and confirmation by cellular morphology and PCR amplification of phylogenetic marker gene. (**B**) BLAST results showed high similarities of hypervariable regions of 16S rRNA gene amplified from two bacterial colonies that were successfully isolated from a CRC patient (M2-PK008A), with those of *P. micra* strains in the RefSeq database.
