# Supplementary material for: Parvimonas micra promotes colorectal tumorigenesis and is associated with prognosis of colorectal cancer patients
Source: Oncogene. 2022 Jul 27;41(36):4200–10. doi: 10.1038/s41388-022-02395-7 (PMC9439953; doi:10.1038/s41388-022-02395-7)
Supplement: Supplementary file 3 — Figure S2 [file 41388_2022_2395_MOESM3_ESM.pdf]

Figure S2

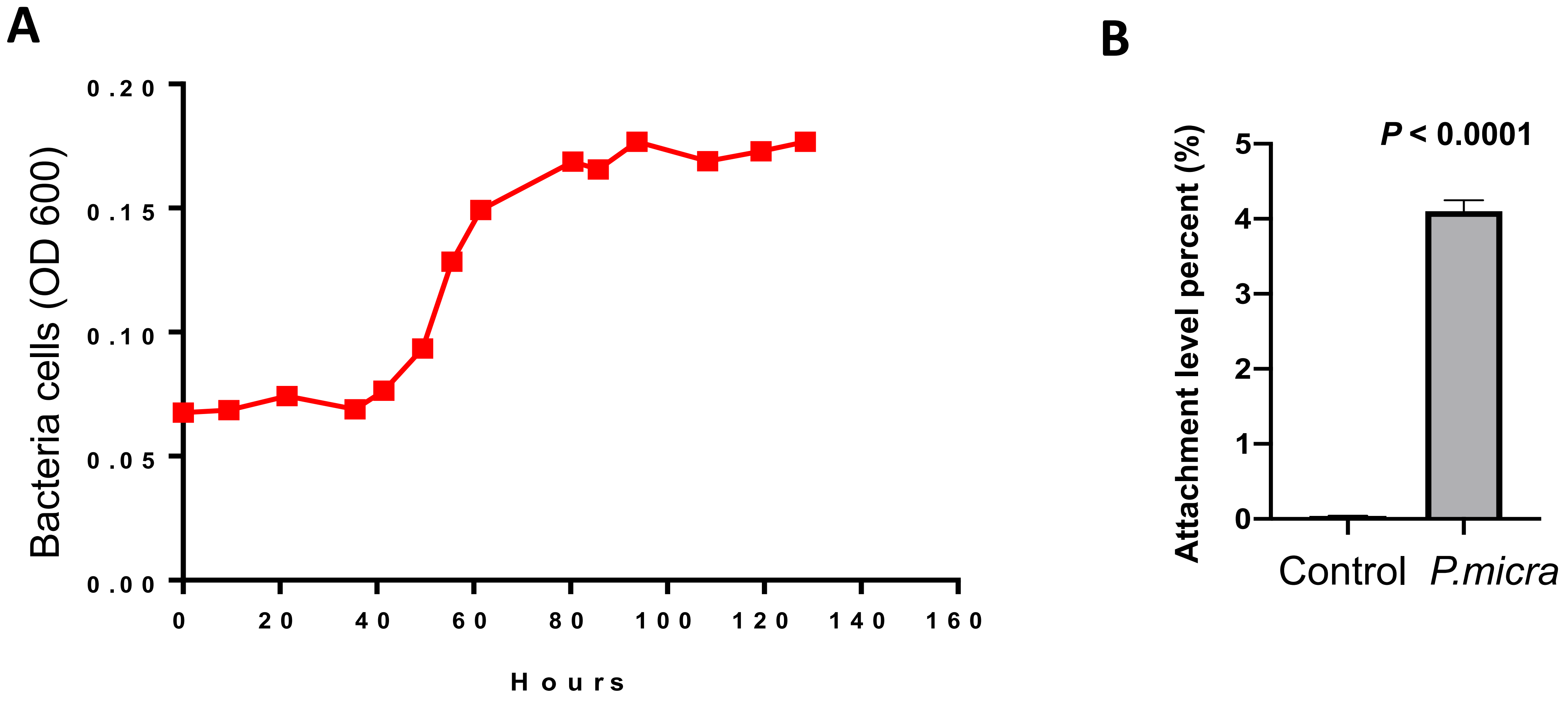

Figure S2. Growth dynamics and colony morphology of clinical *P. micra* strain isolated from a CRC stool sample. (A) Determination of log phase of *P. micra* by growth curve analysis. **(B)** Bacterial attachment assay of *P. micra* on HT29 cells.
