# Supplementary material for: Parvimonas micra promotes colorectal tumorigenesis and is associated with prognosis of colorectal cancer patients
Source: Oncogene. 2022 Jul 27;41(36):4200–10. doi: 10.1038/s41388-022-02395-7 (PMC9439953; doi:10.1038/s41388-022-02395-7)
Supplement: Supplementary file 4 — Figure S3 [file 41388_2022_2395_MOESM4_ESM.pdf]

Figure S3

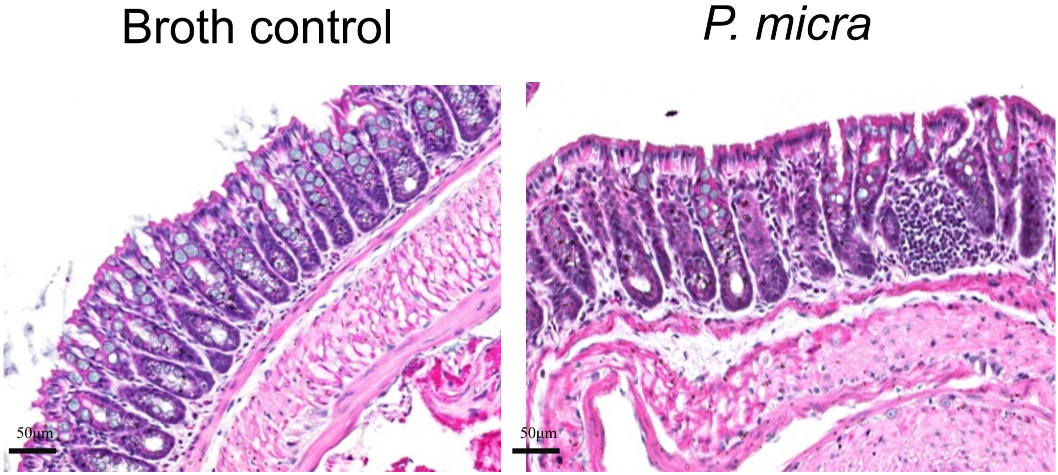

Figure S3. H&E staining of colon in C57BL/6 conventional mice gavaged with *P. micra* or broth control for 9 months.
