# Supplementary material for: Parvimonas micra promotes colorectal tumorigenesis and is associated with prognosis of colorectal cancer patients
Source: Oncogene. 2022 Jul 27;41(36):4200–10. doi: 10.1038/s41388-022-02395-7 (PMC9439953; doi:10.1038/s41388-022-02395-7)
Supplement: Supplementary file 5 — Figure S4 [file 41388_2022_2395_MOESM5_ESM.pdf]

Figure S4

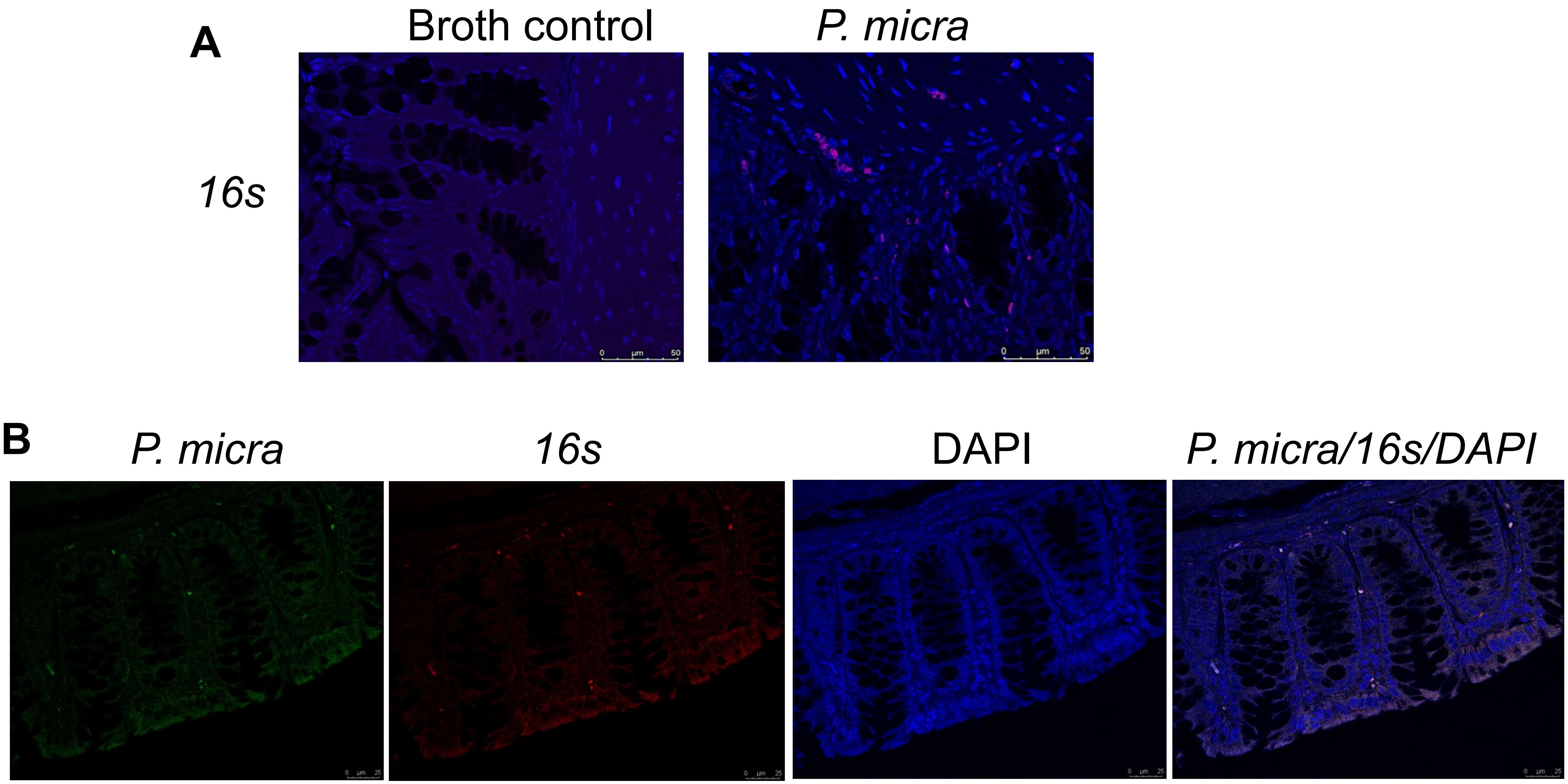

Figure S4. (A) Representative FISH images of colon tissue sections from broth control or *P. micra*-infected mice 36 weeks post-infection (Blue: nuclear; Red: EUB338 universal bacterial probe)*;* (**B**) Representative FISH images of *P. micra*-infected mice colon tissue sections co-stained with EUB338 universal bacterial probe and *P. micra*-specific probe (Blue: nuclear; Green: *P. micra* probe*;* Red: EUB338 universal bacterial probe).
