# Supplementary material for: Parvimonas micra promotes colorectal tumorigenesis and is associated with prognosis of colorectal cancer patients
Source: Oncogene. 2022 Jul 27;41(36):4200–10. doi: 10.1038/s41388-022-02395-7 (PMC9439953; doi:10.1038/s41388-022-02395-7)
Supplement: Supplementary file 7 — Table S1-S2 [file 41388_2022_2395_MOESM7_ESM.docx]

**Table S1. Clinicopathological characteristics and *P. micra* abundance in**

**tumor tissues of colorectal cancer patients.**

|  |  | **Low-abundance**  **(n=99)** | | | **High abundance P value**  **(n=19)** | | | |
| --- | --- | --- | --- | --- | --- | --- | --- | --- |
| **Age** |  | |  |  | |  |  |  |
| <=65 | 28 | | 28.28% |  | | 2 | 10.53% | 0.151 |
| >65 | 71 | | 71.72% |  | | 17 | 89.47% |  |
| **Gender** |  | |  |  | |  |  |  |
| M | 56 | | 56.57% |  | | 8 | 42.11% | 0.317 |
| F | 43 | | 43.43% |  | | 11 | 57.89% |  |
| **TNM clinical stage** | | |  |  | |  |  |  |
| I & II | 46 | | 46.47% |  | | 11 | 57.89% | 0.455 |
| III &  IV | 53 | | 53.54% |  | | 8 | 42.11% |  |
| **Obesity (Asian)** | | |  |  | |  |  |  |
| No | 85 | | 85.86% |  | | 17 | 89.47% | 0.999 |
| Yes | 14 | | 14.14% |  | | 2 | 10.53% |  |
| **Smoking status** | | |  |  | |  |  |  |
| Past | 75 | | 67.82% |  | | 12 | 63.16% | 0.264 |
| Current | 24 | | 54.84% |  | | 7 | 36.84% |  |
| **Diabetes mellitus** | | |  |  | |  |  |  |
| No | 68 | | 68.69% |  | | 14 | 73.68% | 0.789 |
| Yes | 31 | | 31.31% |  | | 5 | 26.32% |  |
| **Oral prescription (4 weeks prior to sampling)** | | | | | | | | |
| No | 14 | | 14.14% |  | | 2 | 10.53% | 0.999 |
| Yes | 85 | | 85.86% |  | | 17 | 89.47% |  |

**Table S2. Primer sequences**

| Gene name | Primer | Sequence |
| --- | --- | --- |
| Mouse TNF-α | Forward | CCCTCACACTCAGATCATCTTCT |
|  | Reverse | GCTACGACGTGGGCTACAG |
| Mouse IL6 | Forward | TCTATACCACTTCACAAGTCGGA |
|  | Reverse | GAATTGCCATTGCACAACTCTTT |
| Mouse IL-17 | Forward | GGAGAGCTTCATCTGTGTCTCTG |
|  | Reverse | TTGAGGGATGATCGCTGCTG |
| Mouse CXCR1 | Forward | ACTGCACCCAAACCGAAGTC |
|  | Reverse | TGGGGACACCTTTTAGCATCTT |
| Mouse β-actin | Forward | GGCTGTATTCCCCTCCATCG |
|  | Reverse | CCAGTTGGTAACAATGCCATGT |
| Human c-myc | Forward | CTGCGACGAGGAGGAGGACT |
|  | Reverse | GGCAGCAGCTCGAATTTCTT |
| Human β-actin | Forward | AGAGCTACGAGCTGCCTGAC |
|  | Reverse | AGCACTGTGTTGGCGTACAG |
| 16s rRNA V1-V4 | Forward | AGAGTTTGATCCTGGCTCAG |
|  | Reverse | CTACCAGGGTATCTAATCC |
| 16s rRNA V6 | Forward | AACGCGAAGAACCTTAC |
|  | Reverse | CGGTGTGTACAAGACCC |
| *Parvimonas. micra* | Forward | AACGACGATTAATACCGCATGAGACC |
|  | Reverse | CTTCCTCCTATGATACCGTCATTA |
| Universe QPCR-Eub341F | Forward | ACTCCTACGGGAGGCAGCAGT |
| Universe QPCR-Eub534R | Reverse | ATTACCGCGGCTGCTGGC |
